# Experiences of Trauma and PTSD Symptoms in Autistic Adolescents: Preliminary Findings

**DOI:** 10.1177/13591045261418319

**Published:** 2026-02-09

**Authors:** Alex Lau-Zhu, Alice M.G. Quinton, James Stacey, Rebecca Roberts-Davis, Myra Cooper, Carmen Chan, Francesca Happé

**Affiliations:** 1 Department of Experimental Psychology, Medical Sciences Division, 6396 University of Oxford, Oxford, UK; 2 Division of Psychiatry, Department of Brain Sciences, Imperial College London, London, UK; 3 Child and Adolescent Mental Health Services, 8955 Oxford Health NHS Foundation Trust, Oxford, UK; 4 Linacre College, Oxford, UK; 5 Social, Genetic, & Developmental Psychiatry Centre, Institute of Psychiatry, Psychology and Neuroscience, 4616 King’s College London, London, UK

**Keywords:** autism, trauma, PTSD, adolescent, maltreatment, mental health

## Abstract

Psychological trauma and post-traumatic stress disorder (PTSD) are under-researched in autistic individuals. We explored the experience of trauma and PTSD symptoms in a sample of autistic adolescents (*n* = 30) aged 10–16 years (without a maltreatment history; 47% female), compared to a group of typically-developing (TD; *n* = 29) and a group of (non-autistic) maltreatment-exposed adolescents (*n* = 28), matched on key demographics. Caregiver reports indicated that a wide range of events were deemed traumatic to autistic adolescents, including those not meeting DSM-5’s Criterion A for trauma for a PTSD diagnosis (e.g., bullying and bereavement). Caregiver- and self-reports converged to show more severe PTSD symptoms, and higher rates of probable PTSD, in autistic adolescents (43–57%) relative to the TD adolescents (7–32%). Symptom severity and rates of probable PTSD were comparable between the autistic and maltreatment-exposed adolescents (50–54%), except that, for autistic adolescents, the index trauma mostly did *not* match DSM-5 criteria, whereas it did for maltreatment-exposed adolescents. This short report’s early findings supports the need for improved assessment of trauma exposure and PTSD symptoms in autistic adolescents. A flexible approach to how trauma is defined in this population may be needed, considering subjective experiences and autism-related processing differences.

## Plain Language Summary

Psychological trauma and post-traumatic stress disorder (PTSD) are not well studied in autistic young people. We looked at experiences of trauma and PTSD symptoms in autistic adolescents aged 10–16 years (*n* = 30; 47% female) who did not have a history of maltreatment. We compared them with two groups of adolescents: typically developing (TD; *n* = 29) and maltreatment-exposed (*n* = 28), with similar ages and other key characteristics. Caregivers reported that autistic adolescents experienced a wide range of events as traumatic, including events that would not usually count as trauma under standard PTSD rules (DSM-5), such as bullying or the death of a loved one. Both caregiver and adolescent reports showed that autistic adolescents had more severe PTSD symptoms and higher rates of probable PTSD (43–57%) compared with TD adolescents (7–32%). Interestingly, the severity of symptoms and rates of probable PTSD in autistic adolescents were similar to those in the maltreatment-exposed group (50–54%). However, while the maltreatment group’s trauma usually met DSM-5 criteria, the traumatic experiences in the autistic group often did not. These early findings highlight the importance of recognising trauma and PTSD symptoms in autistic adolescents, even when the events do not meet standard definitions of trauma. Assessments may need to be more flexible and consider each young person’s subjective experience, as well as differences in how autistic adolescents process events. Overall, the study suggests that autistic adolescents can be highly affected by stressful or upsetting experiences, and mental health support should take their unique perspectives into account.

## Introduction

Psychological trauma and post-traumatic stress disorder (PTSD) have historically been under-researched in autistic people (Hernández-González et al., 2023), but are increasingly recognised as a research priority by the autism community. PTSD appears to affect approximately 14% of autistic children and adolescents and 44% of autistic adults (Quinton et al., 2024; Rumball, 2018), compared to 7.8% developing PTSD by age 18 in non-autistic samples (Lewis et al., 2019) and 6.8–-9.2% of adults in the general population (Goldstein et al., 2016; Kessler & Berglund, 2005). Such estimates overall must be interpreted with caution, as they could be biased/inflated due to methodological issues, including confounding factors, lack of control comparisons, clinical sample recruitment, and reliance on single informants. Only one previous study has considered child self-report (Hoover & Romero, 2019) – an essential perspective as internalising symptoms can often be missed by informants (e.g., Lei et al., 2024). Further, maltreatment history is a major risk for PTSD (Copeland et al., 2007) and frequently co-occurs with autism (Davidson et al., 2022; Gajwani & Minnis, 2023) but is seldom accounted for.

What is perceived as “trauma” may be different for autistic people, and strict definitions of trauma could risk poor identification and support (Brewin et al., 2019; van den Berg et al., 2017). Rumball and colleagues (2020) found that over 40% of autistic adults met criteria for probable PTSD in response to events deemed as subjectively “traumatic” (e.g., bullying, bereavement, and relationship difficulties), but which did *not* meet the definition of trauma required for a PTSD diagnosis in the DSM-5 Criterion A – “exposure to actual or threatened death, serious injury, or sexual violence” (APA; American Psychiatric Association, 2013). Similarly, autistic adults and caregivers of autistic children, interviewed by Kerns and colleagues (2022), described various experiences as traumatic outside of standardised measures, including ones which reflected a conflict between autistic characteristics and the environment (e.g., sensory traumas). There has yet to be a quantitative exploration of PTSD symptoms associated with such experiences of trauma exposure in autistic individuals during adolescence, a period of peak mental health vulnerability (Solmi et al., 2021).

Our aim was to build on this important prior work to raise the possibility that our understanding of PTSD in autistic adolescents may need to consider a wider range of stressors. Drawing on a small yet unique dataset, we conducted an exploration of experiences of trauma and PTSD symptoms and, for the first time in autistic adolescents, distinguished DSM-5 versus no-DSM-5-qualifying events, combined self- and caregiver-reports, and explored selected individual differences previously linked to autistic cognition (Rumball, 2018). Our autism group had no maltreatment history, and as trauma/PTSD were not mentioned in recruitment materials, we somewhat minimised potential inflation of probable PTSD rates. Contrasting this group with a demographically-matched control group and a maltreatment-exposed (non-autistic) group provided preliminary indications of how autism may uniquely relate to PTSD risk.

## Methods

### Participants

Eighty-seven adolescents (and their caregivers) took part during Covid-19 pandemic (September 2021–April 2022) in a wider study on cognition and mental health (Lau-Zhu, Chan, et al., 2024; Lau-Zhu, Stacey, et al., 2024). The study comprised three groups of adolescents aged 10–16 years. The autism group (*n* = 30) met DSM/ICD criteria for autism spectrum disorder (ASD) (verified with diagnostic documentation provided by keyworkers/caregivers) and no maltreatment history (abuse/neglect). The TD group (*n* = 29) had neither an ASD diagnosis nor a maltreatment history. The maltreatment group (*n* = 28) had maltreatment histories but no ASD diagnoses nor first/second-degree relatives with ASD. All adolescents had no diagnosis of learning disability and were required to read English. Consent was obtained from adolescents aged 16 and all parents/caregivers, and assent was obtained from adolescents aged below 16. See Lau-Zhu, Chan, et al. (2024) for full recruitment strategy and Table 1 for further sample characterisation. The West Midlands – Solihull Research Ethics Committee (21/WM/0125) provided ethical approval. See Supplemental Materials for additional details on recruitment and selection strategy.

**Table 1.** Background Variables Including Demographics and Clinical Measures, by Group

	Autism (*n* = 30)	Maltreatment (*n* = 28)	TD (*n* = 29)	Group comparisons^a^ (*p*-value)
Age, years: mean (*SD*)	12.47 (1.98)	13.57 (2.08)	12.93 (1.98)	.118
Sex (at birth)^b^: *n* females (%)	14 (47%)	17 (61%)	15 (52%)	.557
Ethnicity; *n* white (%)	28 (93%)^c^	19 (68%)^d^	18 (62%)^d^	.013
Socioeconomic status (SES)^e^, parental education: *n* (%) at university level	21 (70%)	13 (54%)	19 (68%)	.437
Diagnoses of neurodevelopmental disorders (not autism)^f^: *n* (%)	13 (43%)^c^	2 (7%)^d^	0^d^	<.001
Diagnoses of PTSD: *n* (%)	2 (6%)	1 (4%)	0	NA^g^
Diagnoses of emotional disorders (others)^h^: *n* (%)	8 (27%)^c^	2 (7%)^d^	0^d^	.004
Medication, yes: *n* (%)	14 (48%)^c^	1 (4%)^d^	0^d^	<.001
Talking therapy, yes: *n* (%)	13 (43%)^c^	18 (64%)^c^	0^d^	<.001
Cognitive behavioural intervention	6	5	0	
Evidence-based trauma-focused intervention	1	1	0	
Recruitment source				<.001
Healthcare	14	5	0	
Social care	0	23	0	
Community	16	0	29	

*Note.* TD = typical development (i.e., non-autistic and non-maltreatment control group).

^a^Overall group comparisons were done using chi-square tests for categorical variables and with one-way ANOVA for continuous variables (*p*-values).

^b^One autistic participant (female assigned at birth) identified as transgender.

^c,d^Within each row, group values that have different superscript letters (a, b) are significantly different from each other (*p* < .05). Group values sharing the same letter are not significantly different. Independent sample t-tests were used for continuous variables and chi-square tests for categorical variables.

^e^Missing data were as follows: maltreatment, n = 4; TD*, n* = 1.

^f^Included ADHD, Tourette’s syndrome, dyspraxia, dyscalculia, dyslexia, and sensory processing disorder.

^g^Observed frequencies were <5 and thus statistical comparisons were not run

^h^Included obsessive compulsive disorder, body dysmorphic disorder, depressive disorder, generalised anxiety disorder.

### Measures

See Supplemental Materials for additional details on measures.

#### Trauma Exposure

The *Child and Adolescent Trauma Screen – Caregiver Version* (CATS; Sachser et al., 2017) has 14 items assessing potentially traumatic events based on DSM-5 criteria, including physical harm, sexual assault and natural disasters. To assess non-DSM-5 traumas, two additional questions (following Rumball et al., 2020) asked: “any other events that have happened to your child that they would consider stressful, scary, traumatic or extremely unpleasant?” and/or “… causing nightmares or memories of the event(s) to come back into their mind, when they did not want to think about them?”. Caregivers were asked to identify a specific index event before reporting PTSD symptoms in the CATS, which was also checked to see if it matched the DSM-5 criteria.

#### PTSD Symptomatology

The CATS (Sachser et al., 2017) provides caregiver report for 20 items measuring symptom frequency on intrusion, avoidance, negative mood/cognition, and hyperarousal, with a cut-off score for probable PTSD of ≥ 21 (Nilsson et al., 2020). A novel exploratory item on vocal/motor tics was added after consultation with caregivers of autistic adolescents. The 8-item *Children’s Revised Impact of Event Scale* (CRIES; Perrin et al., 2005) was selected for adolescent self-report due to its brevity to minimise completion burden. This covers symptoms of re-experiencing and avoidance, with a cut-off score for probable PTSD of ≥ 17 (Perrin et al., 2005). Symptoms in the CRIES are reported without the need for the adolescent to self-describe the index trauma.

#### Individual Differences

Autistic traits were measured with the *Social Communication Questionnaire – Current Form* (Rutter et al., 2003). Cognitive traits considered were executive functioning, using the *Dysexecutive Questionnaire – Children* (Emslie et al., 2003); general cognitive ability, using the abbreviated 9-item form of the *Raven’s Standard Progressive Matrices Test* (Bilker et al., 2012); and trait imagery vividness, using the *Plymouth Sensory Imagery Questionnaire* (Andrade et al., 2014).

### Procedure

Adolescents and their caregivers completed separate online questionnaires. These were reviewed for suitability (ease of comprehension and perceived burden) by autistic/maltreatment-exposed adolescents and caregivers/practitioners. Recruitment materials did not mention trauma/PTSD in autism. See Supplemental Materials for additional details.

### Analytical Approach

Data were checked for normality and outliers. Group differences were assessed with ANOVAs, independent-sample/Welch’s t-tests (for continuous variables), and Chi-square/Fisher’s exact tests (for categorical variables, e.g., yes vs. no experience of each traumatic event separately). Associations were assessed with Pearson’s/Spearman’s correlations (for continuous variables), and Chi-square tests (for categorical variables). Parametric tests for nonnormal data are reported when these converged with nonparametric tests, unless otherwise stated. Fisher’s Exact Tests were used to compare correlation sizes. Qualitative descriptions of traumatic events (unlinked to group membership) were double-coded (by ALZ and RRD; yes vs. no) as whether each met DSM-5 Criterion A required for a PTSD diagnosis (APA, 2013), with almost perfect agreement, Cohen’s kappa = 0.91 (Altman, 1991).

## Results

### Trauma Exposure

#### DSM-5 Traumatic Events

**Figure 1.**
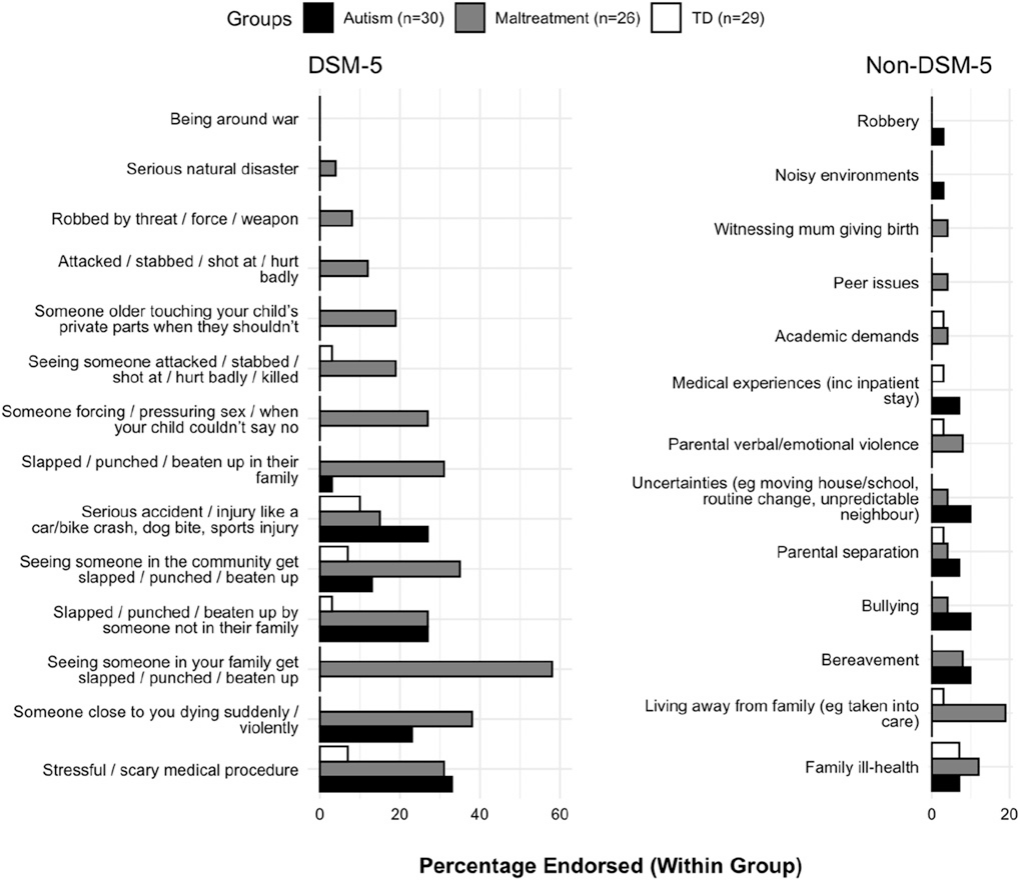
Percentage of DSM-5 and non-DSM-5 traumatic events (within groups) reported by caregivers

Relative to the TD group, both the autism and maltreatment groups reported higher incidence of physical assaults, traumatic bereavements and stressful medical procedures (*p*’s < .05; Figure 1). The maltreatment group additionally reported higher incidence of familial physical violence, sexual harm, or community violence (*p*’s < .05). The median number of DSM-5 events endorsed was 1 (IQR = 2) for autistic adolescents; 3 (IQR = 4) for maltreatment-exposed adolescents; and 0 (IQR = 0) for TD adolescents.

#### Non-DSM-5 Traumatic Events

Caregivers of 17 autistic (56.7%), 18 maltreatment-exposed (69.2%), and 8 TD (27.6%) adolescents, reported an event that the adolescents found traumatic, but which did not match DSM-5 criterion (Figure 1). The most reported events were bullying, bereavements, and events involving change/uncertainty in the autism group; and events related to out-of-home care in the maltreatment-exposed group.

#### Overlap in Traumatic Events

Each trauma-exposed adolescent across groups was categorised based on event types endorsed (i.e., DSM-5-only, non-DSM-5-only, or both). Autistic (*n* = 14/30, 46.7%) and maltreatment-exposed adolescents (*n* = 15/26, 57.7%) reported both DSM-5 and non-DSM-5 traumas simultaneously more frequently than TD adolescents (*n* = 2/29, 6.9%; *p*’s < .002). Autistic, maltreatment-exposed and TD groups were compared by their frequencies of reporting DSM-5-only (*n*’s = 2–8; 6.9–32.0%) or non-DSM-5-only events (*n*’s = 2–5, 7.7–17.2%) and did not differ significantly (*p*’s > .05).

### PTSD Symptomatology

According to caregiver- and self-reports (Table 2), the autistic and maltreatment-exposed adolescents reported more severe PTSD symptoms overall compared to TD (*d*’s = 0.55–1.04). Both autistic (by self-report only) and maltreatment-exposed (by both informants) adolescents reported significantly more intrusion symptoms. They also reported significantly more avoidance, hyperarousal, and negative mood/cognition symptoms (by caregiver report only in both groups). Symptom severity did not significantly differ between the autism and maltreatment groups (*d*’s = 0.01–0.25), but the autistic group reported relatively more tics in response to the index event.

**Table 2.** PTSD Symptoms by Caregiver and Self Report, by Group

	Autism	Maltreatment	TD
CATS - caregiver reports available^a^	*n* = 23	*n* = 26	*n* = 10^a^
Total (max = 60), *mean (SD)*^b^	21.39 (11.52)^c^	25.04 (17.46)^c^	10.20 (9.98)^d^
Intrusion (max = 15), *mean (SD)*	4.13 (3.43)^c,d^	5.42 (4.60)^c^	2.23 (1.89)^d^
Avoidance (max = 6), *mean (SD)*	2.52 (2.27)^c^	2.65 (2.31)^c^	0.70 (0.95)^d^
Negative mood & cognitions (max = 21), *mean (SD)*	7.21 (4.66)^c^	9.04 (7.01)^c^	3.20 (3.99)^d^
Hyperarousal (max = 18), *mean (SD)*	7.65 (4.35)^c^	7.92 (5.15)^c^	4.00 (4.50)^d^
Tics (novel item; max = 3), *median (IQR)*^e^	1 (2)^c^	0 (2)^d^	0 (0)^d^
Probable PTSD, *n (%)*^b^	13 (43%)^c^	13^c^ (50%)^c^	2^d^ (7%)^d^
CRIES - self-reports available^a^	*n* = 28	*n* = 28	*n* = 28
Total (max = 40), *mean (SD)*	19.21 (12.18)^c^	19.07 (13.44)^c^	12.46 (10.43)^d^
Intrusion (max = 20), *mean (SD)*	9.25 (6.39)^c^	9.36 (7.30)^c^	5.79 (5.12)^d^
Avoidance (max = 20), *mean (SD)*	9.96 (6.98)^c^	9.71 (7.08)^c^	6.68 (5.74)^c^
Probable PTSD, *n (%)*	16 (57%)^c^	15 (54%)^c^	9^d^ (32%)^d^

*Note.* CATS = Child and Adolescent Trauma Screen; CRIES = Child Revised Impact of Event Scale; PTSD = post-traumatic stress disorder; TD = typical development (i.e., non-autistic and non-maltreatment control group).

^a^PTSD symptoms were reported in the CATS only when a traumatic event was identified by the caregiver, but in the CRIES for all adolescents as the latter does not require them to explicitly describe the index trauma. Thus, data were more available for self- rather than caregiver-reports.

^b^Scoring excludes the novel item.

^c,d^Within each row, group values that have different superscript letters (c, d) are significantly different from each other (*p* < .05). Group values sharing the same letter are not significantly different.

^e^Endorsement of this item per group: autism, *n* = 16; maltreatment, *n* = 5; typical development, *n* = 0.

Reports from different respondents on overall symptom severity showed large and medium correlations in the maltreatment and autism group, respectively. The correlation between informant reports for intrusion (but not avoidance) was significantly bigger for the maltreatment-exposed than the autism group. We did not compute the corresponding correlation in the TD group because caregiver reports were insufficient (*n* = 10), reflecting low caregiver-reported trauma exposure in this group (see Table 2).

**Figure 2.**
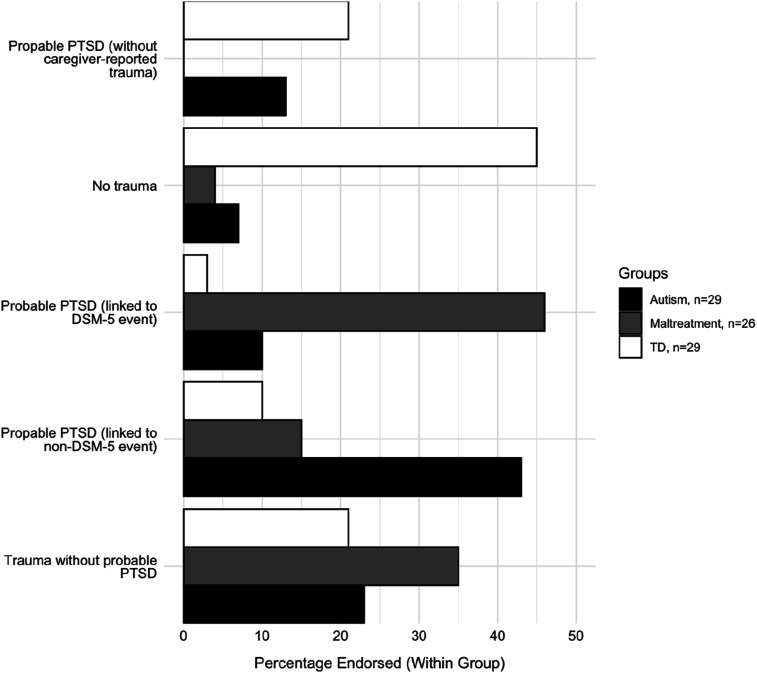
Percentage of cases endorsing trauma exposure and/or crossing cut-offs for probable PTSD, per group. *Note*. Probable PTSD classification is determined based on crossing cut-off on CATS and/or CRIES

Autistic and maltreatment-exposed adolescents showed higher rates of probable PTSD than TD (Figure 2). Based on their caregiver’s selection of the index trauma, probable PTSD was mostly in response to non-DSM-5 events in autistic adolescents but was mostly in response to DSM-5 events in maltreatment-exposed adolescent. The association between groups and if the trauma was DSM-5 event or not was significant (*p* < .001).

See Supplemental Materials for detailed results.

### Exploring Individual Differences

Across groups, more severe PTSD symptomatology was positively and significantly correlated with executive dysfunction and female sex. These correlations were not significant when restricting to each group only, likely owing to reduced power (except for the correlation with sex in the maltreatment group). Correlations (across and within groups) were not significant between PTSD symptoms and remaining individual difference variables (i.e., autistic traits, cognitive ability, and trait imagery) and between exposure to DSM-5/non-DSM-5 traumas and sex. See Supplemental Materials for detailed results.

## Discussion

We report, for the first time, preliminary high rates of PTSD symptoms in a sample of autistic adolescents in response to events not qualifying DSM-5’s Criterion A for trauma. These included bullying, bereavement, events involving change/uncertainty, and family stresses, echoing findings in autistic adults (Kerns, Lankenau, et al., 2022; Rumball et al., 2020) and qualitative data from caregivers of autistic children (Kerns, Lankenau, et al., 2022). While our sample size was modest, findings were of medium-to-large effects and relatively consistent across informants.

Our sample rate of 43–57% probable PTSD for autistic adolescents is comparable to data with autistic adults (47%; Rumball et al., 2020), but higher than in our demographically-matched TD group (7–32%), and recent estimates with autistic (14%; Quinton et al., 2024) and non-autistic youth (1.6%; Kessler et al., 2012). However, a meta-analysis with strict inclusion criteria only found 0–3.6% PTSD diagnostic rates in autistic individuals (Lai et al., 2019). Also, despite our tentative high estimates, only two autistic participants reported a formal PTSD diagnosis (Table 1). Formal diagnoses, unlike symptom measures, may underestimate the impact of trauma in autistic populations. Alternatively, questionnaire measures may be oversensitive. Accurate estimates will require sensitive and specific PTSD assessment tools in autistic adolescents (including validation of existing tools), to avoid risks of “conceptual bracket creep” and overmedicalisation (McNally, 2003). Autism-specific manifestations (beyond exacerbation of existing traits) may also need considering (Kerns & Kendall, 2012; Kerns, Robins, et al., 2022), such as stereotyped behaviours, as hinted by the present study’s novel stakeholder-developed item.

Brewin and colleagues (2019) have argued that strict definitions of trauma could hamper diagnoses – most autistic adolescents’ symptoms in our study were anchored to a caregiver-reported *non*-DSM-5 event rather than a traditional DSM-5 events, as in the maltreatment group (Figure 2), despite comparable symptom levels. While the definition of “trauma” remains controversial (McNally, 2003), emerging data (Kerns, Lankenau, et al., 2022; Rumball et al., 2020), alongside our clinical and empirical observations, encourage us to better attend to autistic lived experiences of “trauma”.

Autistic social communication differences may result in PTSD symptoms being missed by family and/or professionals, leading to potential under-recognition (Haruvi-Lamdan et al., 2018; Kerns et al., 2015). We also found lower concordance between self- and caregiver reports in the autistic relative to the maltreatment-exposed group, particularly on intrusion symptoms such as flashbacks/nightmares, often “invisible” to caregivers.

For individual differences, our finding of higher levels for (autistic) females is consistent with population findings (Olff, 2017). Executive dysfunction (but not autistic traits or cognitive abilities) was positively associated with more symptoms, aligning with adult data (Rumball et al., 2021). Unlike earlier suggestions (Bryant & Harvey, 1996), trait imagery ability did not seem important, but emotional aspects of imagery were not assessed (Di Simplicio et al., 2019; Iyadurai et al., 2019; Lau-Zhu et al., 2023). Potential mechanisms for (increased) PTSD symptoms in autism should be further investigated (e.g., detail-focused/sensory processing; see Hoover, 2015; Peterson et al., 2019; Rumball et al., 2021).

To better understand and assess PTSD symptoms in autistic adolescents, we may need to consider a less restrictive trauma definition (Brewin et al., 2019), multiple informants (including self-reports), and subjective experiences. Indeed, cognitive theories (Ehlers & Clark, 2000) and recent findings (Danese & Widom, 2020) highlight a subjective “sense of threat” as core to trauma. The International Statistical Classification of Diseases and Related Health Problems states that traumatic events within a PTSD diagnosis must be “extremely threatening or horrific” (ICD-11; World Health Organization, 2019), and does not impose standardised event types. As the debate of what is “trauma” in autistic people remains ongoing, a neurodevelopmentally-informed, formulation-based approach, which integrates autistic cognition and meaning-making (e.g., Stark et al., 2021), could be useful.

As evidence-based psychological interventions are acceptable and effective for autistic adolescents (Quinton et al., 2024; Rumball, 2018), these should be adapted appropriately (Peterson et al., 2019) and empirically tested in autistic individuals in relation to distress linked to non-DSM-5 traumas. Intrusive imagery symptoms may also benefit from focal approaches, such as using visuospatial tasks (Lau-Zhu & Chan, 2025; Lau-Zhu et al., 2019, 2021; Rackham & Lau-Zhu, 2021), which could harness common autistic strengths/preferences (Muth et al., 2014).

In sum, this brief report’s early findings – from a community sample during a pandemic period – add to the emerging literature suggesting that current definitions of psychological trauma may not fully capture autistic experiences (Kerns, Lankenau, et al., 2022; Rumball et al., 2020). A better understanding of autism-related processing differences and their impact of trauma will be critical. Future studies should use improved tools, more diverse and representative samples across contexts, and closely involve the autism community.

## Supplemental Material

Supplemental Material - Experiences of Trauma and PTSD Symptoms in Autistic Adolescents: Preliminary Findings Supplemental Material for Experiences of Trauma and PTSD Symptoms in Autistic Adolescents: Preliminary Findings by Alex Lau-Zhu, Alice Quinton, James Stacey, Rebecca Roberts-Davis, Myra Cooper, Carmen Chan, Francesca Happé in Clinical Child Psychology and Psychiatry

## Data Availability

The data that support the findings of this study are available from the corresponding author upon reasonable request.*
