# Supplementary material for: Experiences of Trauma and PTSD Symptoms in Autistic Adolescents: Preliminary Findings
Source: Clin Child Psychol Psychiatry. 2026 Feb 9;31(2):614–27. doi: 10.1177/13591045261418319 (PMC12992638; doi:10.1177/13591045261418319)
Supplement: Supplemental Material - Experiences of Trauma and PTSD Symptoms in Autistic Adolescents: Preliminary Findings [file sj-pdf-1-ccp-10.1177_13591045261418319.pdf]

Supplemental Materials:

Experiences of Trauma and PTSD symptoms in Autistic Adolescents: Preliminary Findings

Additional Methods

Participants

Participants were recruited from several sources. Autistic participants were recruited from local mental health services and community advertisements such as charities; TD participants from community advertisements (e.g., social media and university advertisements) and word of mouth (e.g., asking families to share information); and maltreatment-exposed participants from local mental health and social care services.

The process for verifying suitability was based on the recruitment source, prioritising keyworkers whenever possible as they had access to service records. For autistic participants, the presence of autism diagnoses and the absence of maltreatment were determined by keyworkers (when referred from services) or parents (when referred from the community). For maltreatment-exposed participants, the presence of maltreatment and the absence of autism diagnoses were determined by keyworkers (as all participants from this group were recruited from services). The types of maltreatment histories reported included sexual abuse (n = 6), physical abuse (n = 9), emotional abuse (n = 15) and neglect (n = 23), with many experiencing more than maltreatment type (n = 19). For TD participants, verification on autism diagnoses and maltreatment history were determined by parents/caregivers.

Across groups, maltreatment was further ruled out by prior contact with social services, and autism diagnoses were further ruled out by presence of first-/second-degree relatives with a diagnosis.

Note there was no financial compensation and participant was completely voluntary. These study conditions would have minimised the risk of misreporting from parents/caregivers, given that the main incentive to take part was just to support youth mental-health research.

Procedure

Participants were given a unique ID to access the online surveys, one for the adolescent and one for the caregiver/parent. Response times were reviewed for any implausible short completion times but none were identified. Note the survey was not paid, so there was minimal incentive to produce inauthentic responses or rush through the survey.

Measures

The **Child and Adolescent Trauma Screen** (Sachser et al., 2017) covers symptoms of Intrusion (e.g., “bad dreams related to a stressful event”), avoidance (e.g., “trying not to remember, talk about or have feelings about a stressful event”), negative mood/cognition (“having very negative emotional states [afraid, angry, guilty, ashamed])”, and hyperarousal (e.g., “being overly alert or on guard”). Following consultation with parents/supporters of autistic adolescents, an exploratory item was added: “Sudden onset or increased occurrence of repeated vocal ‘tics’ (e.g., phrases, word patterns, e.g.), and/or motor ‘tics’ (e.g., flapping arms or hands)”. Response options use 4-point Likert scale anchored by “never”, “once in a while”, “half the time”, and “almost always”. Index trauma was selected by the caregiver based on their response to “which one is bothering the child most now?”. Symptoms were assessed only for those who endorsed a traumatic event. Total score on the CATS did not include this novel exploratory item. The internal consistencies per subscale (Cronbach’s alpha) was acceptable to strong in the autism group (n = 23, intrusion = 0.79, avoidance = 0.83, hyperarousal = 0.75, negative cognitions & mood = 0.74), and good to excellent in the maltreatment group (n = 26, intrusion = 0.91, avoidance = 0.86, hyperarousal = 0.87,

negative cognitions & mood = 0.93). These were not computed for the TD sample due to the small sample size of caregivers who endorsed a DSM-5 traumatic event for their child (n = 10).

The **Children's Revised Impact of Event Scale** (CRIES; Perrin et al., 2005) covers symptoms of re-experiencing (e.g., "do pictures of what happened pop into your mind?") and avoidance (e.g. "do you try to avoid talking about the event?"). Response options use 4-point Likert scale anchored by "not at all", "rarely", "sometimes" and "often". Index trauma was not required to complete the questionnaires. The internal consistencies per subscale (Cronbach's alpha) was acceptable in the autism group (n = 28, intrusion = 0.79, avoidance = 0.77), good in the maltreatment group (n = 28, intrusion = 0.86, avoidance = 0.87), and good in the TD group (n = 28, intrusion = 0.80, avoidance = 0.82).

The **Social Communication Questionnaire – Current form** (SCQ; Rutter et al., 2003) is a screening questionnaire for autism spectrum disorders (ASD) in childhood The Current form (over the most recent three-month period) was chosen rather the Lifetime form for comparability across the three groups, as many caregivers (e.g., adoptive and foster carers) of maltreatment-exposed adolescents would not necessarily know the adolescent's developmental history. Total scores range 0-39. Internal consistency (Cronbach's alpha) in this study was acceptable (autism, n = 30, 0.77; maltreatment, n=29, 0.76; TD, n = 29, 0.73).

The Abbreviated 9-item form of the **Raven's Standard Progressive Matrices Test** (Bilker et al., 2012) was used as a proxy for general cognitive ability in adolescents (Bone et al., 2021). It has been shown to be highly predictive of the original longer form (Raven, 2000). As this is a performance-based task, the notion of internal consistency is not applicable.

The **Dysexecutive Questionnaire – Children** (Emslie et al., 2003) assesses difficulties associated with emotional/personality, motivational, behavioural, and cognitive aspects of

executive functioning. Items scoring range 0-4 (from “never” to “very often”). Internal consistency (Cronbach’s alpha) in this study was acceptable to excellent (autism, n = 27, 0.90; maltreatment, n = 25, 0.93; TD, n = 28, 0.73).

The **Plymouth Sensory Imagery Questionnaire** probes the ability to generate vivid imagery in relation to scenarios presented across different senses, including visual, auditory and tactile modalities (Andrade et al., 2014). Items scoring range is 0 to 10. Internal consistency (Cronbach’s alpha) in this study was excellent (autism: n = 27; 0.96; maltreatment group, n = 25; 0.95; TD group, n = 28, 0.85).

Additional Results

PTSD Symptomatology

Relative to the TD group, the autism group reported significantly more symptoms in the CATS, for total scores, $t(31) = 2.66, p = .012, d = 1.01$, avoidance, $t(31) = 3.25, p = .003, d = 0.92$, negative cognitions/mood, $t(31) = 3.27, p = .024, d = 0.90$, and arousal, $t(31) = 2.19, p = .036, d = 0.83$, and marginally for intrusions, $t(28.95) = 1.97, p = .059, d = 0.60$, as well as significantly more symptoms in the CRIES for total scores, $t(54) = 2.23, p = .030, d = 0.60$ and intrusions, $t(54) = 2.24, p = .029, d = 0.60$, but not avoidance, $t(54) = 1.92, p = .060, d = 0.51$. The autism group also reported significantly more tics, $U = 35.00, p < .001, r = 0.60$.

Relative to the TD group, the maltreatment group reported more symptoms in the CATS, for total scores, $t(28.47) = 3.19, p = .003, d = 0.94$, intrusions, $t(33.72) = 2.89, p = .007, d = 0.77$, avoidance, $t(33.73) = 3.59, p = .001, d = 0.96$, negative cognitions/mood, $t(28.56) = 3.12, p = .004, d = 0.92$, arousal, $t(18.67) = 2.25, p = .037, d = 0.79$, as well as more symptoms in the CRIES for total scores, $t(50.86) = 2.06, p = .045, d = 0.55$, intrusions, $t(48.41) = 2.12, p = .039, d = 0.57$, but not avoidance, $t(51.77) = 1.76, p = .084, d = 0.47$. However, the maltreatment group did not report significantly more tics, $U = 105.00, p = .142, r = 0.25$.

The autism and maltreatment group did not report significant differences in symptoms of CATS for total scores, intrusions, avoidance, negative cognitions/mood, arousal, t's < 1.10, p's > .275, d's < 0.32, or symptoms of CRIES for total scores, intrusions, and avoidance, t's < 1, p's > .895, d's < 0.04. However, the autism group reported significantly more tics, U = 150.50, p < .001, r = 0.48.

For overall symptom severity, both self- and caregiver-reports significantly correlated, with strong correlation for the maltreatment group, $r(26) = 0.73$, p <.001, n = 26, but a moderate correlation in the autism group, $r(22) = 0.45$, p = .037, n = 22. Correlation was not computed for the TD group due to small sample size, n = 9. Analyses were also split by subscales. In the autism group, informant reports significantly correlated for avoidance, r = 0.46, p = .030, but not intrusions, r = 0.30, p = .183. In the maltreatment group, informant reports significantly correlated for avoidance, r = 0.56, p = .003, and intrusions, r = 0.82, p < .001. The size of the correlation was statistically bigger in the maltreatment group compared to the autism group for intrusions, p = .003, but not avoidance, p = .331.

Individual Differences

Executive dysfunction. Across groups, more severe PTSD symptomatology was positively and significantly correlated with executive dysfunction (CRIES: r = 0.25, p = .025, N = 82; CATS: r = 0.46, p < .001, N = 59). These correlations were not significant when restricting to the autism group only (CRIES: r = 0.10, p = .622, n = 28; CATS: r = 0.38, p = .075, n = 23) or the maltreatment group only (CRIES: r = 0.11, p = .609, n = 26; CATS: r = 0.36, p = .068, n = 26). For the TD group, the correlation was not significant for CRIES (r = 0.05, p = .819, n = 28), but significant for CATS (r = 0.67, p = .034, n = 10) but which was based on a very small sample and would not survive familywise error correction (.05/6=.008).

Autistic traits. Correlations between PTSD symptoms and autistic traits were not significant across groups (CRIES: $r = 0.17$, $p = .123$, $N = 82$; CATS: $r = 0.24$, $p = .073$, $N = 59$), or within each group, including autism (CRIES: $r = -0.08$, $p = .674$, $n = 28$; CATS: $r = -0.14$, $p = .516$, $n = 23$), maltreatment (CRIES: $r = 0.11$, $p = .599$, $n = 26$; CATS: $r = 0.37$, $p = .061$, $n = 26$), and TD (CRIES: $r = 0.02$, $p = .937$, $n = 28$; CATS: $r = 0.61$, $p = .062$, $n = 10$).

Cognitive ability. Correlations between PTSD symptoms and cognitive ability were not significant across groups (CRIES: $r = -0.09$, $p = .430$, $N = 82$; CATS: $r = -0.19$, $p = .187$, $N = 55$) or within the autism group (CRIES: $r = -0.26$, $p = .185$, $n = 27$; CATS: $r = -0.26$, $p = .248$, $n = 21$). In the maltreatment group, the correlation was significant for CRIES ($r = 0.43$, $p = .027$, $n = 27$), which would not survive familywise error correction (.05/6=.008), and not for CATS ($r = 0.24$, $p = .242$, $n = 25$). In the TD group, the correlation was significant for CATS ($r = -0.78$, $p = .013$, $n = 9$), which is based on a very small sample size and would survive familywise error correction, and not for CRIES ($r = -0.09$, $p = .651$, $n = 28$).

Trait imagery. Correlations between PTSD symptoms and trait imagery were not significant across groups (CRIES: $r = 0.07$, $p = .521$, $N = 82$; CATS: $r = -0.10$, $p = .482$, $N = 55$), or within each group, including autism (CRIES: $r = 0.16$, $p = .416$, $n = 27$; CATS: $r = -0.20$, $p = .381$, $n = 21$), maltreatment (CRIES: $r = 0.08$, $p = .699$, $n = 27$; CATS: $r = 0.02$, $p = .942$, $n = 25$), and TD (CRIES: $r = -0.07$, $p = .711$, $n = 28$; CATS: $r = -0.66$, $p = .055$, $n = 9$).

Sex (assigned at birth). Across groups, more severe PTSD symptomatology was positively and significantly correlated with female sex (CRIES: $r = 0.31$, $p = .004$, $N = 84$; CATS: $r = 0.32$, $p = .015$, $N = 59$). These correlations were still significant when restricting to the maltreatment group only (CRIES: $r = 0.63$, $p < .001$, $n = 28$; CATS: $r = 0.42$, $p = .035$, $n = 26$), but not in the autism group only (CRIES: $r = 0.17$, $p = .390$, $n = 28$; CATS: $r = 0.29$, $p = .177$, $n = 23$) or the TD group only (CRIES: $r = 0.10$, $p = .609$, $n = 28$; CATS: $r = 0.26$, $p = .478$, $n = 10$). Female sex was not significantly correlated with more exposure to DSM-5

traumatic events across groups (*r* = -0.07, *p* = .516, *N* = 85) or within each group, namely autism (*r* = -0.23, *p* = .232, *n* = 30), maltreatment (*r* = -0.18, *p* = .384, *n* = 26), and TD (*r* = -0.01, *p* = .972, *n* = 29). Female sex was not significantly associated with exposure to nonDSM-5 traumatic events across groups (*p* = .080) or within the maltreatment group (*p* = .226) or TD group (*p* = .129), but this was significant in the autism group (*p* = .028), which would not survive familywise error correction (.05/3=.017).
